# Supplementary material for: Distinct effects of different matrix proteoglycans on collagen fibrillogenesis and cell-mediated collagen reorganization
Source: Sci Rep. 2020 Nov 4;10:19065. doi: 10.1038/s41598-020-76107-0 (PMC7642422; doi:10.1038/s41598-020-76107-0)
Supplement: Supplementary file 1 — Supplementary Information. [file 41598_2020_76107_MOESM1_ESM.docx]

**Supplementary Materials**

**Distinct effects of different matrix proteoglycans on collagen fibrillogenesis and cell-mediated collagen reorganization**

Dongning Chen^1,2,3^, Lucas R. Smith^4^, Gauri Khandekar^4^, Pavan Patel^5^, Christopher K. Yu^1^, Kehan Zhang^6^, Christopher S. Chen^2,6^, Lin Han^5^, Rebecca G. Wells^1,2,3,4,7^


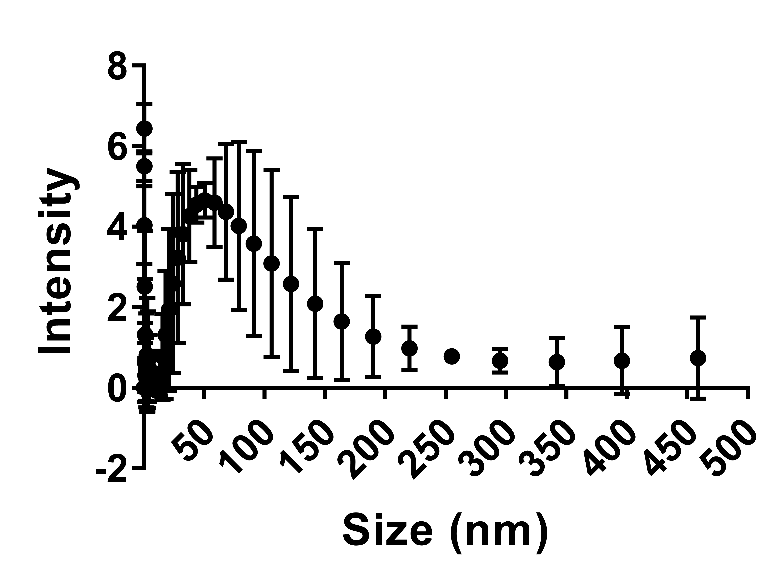


**Supplementary Fig. 1.** The size distribution of chondroitin sulfate tested by dynamic light scattering. CS sodium salt isolated from bovine cartilage (Sigma) has a distribution of sizes ranging from 10-150 nm.


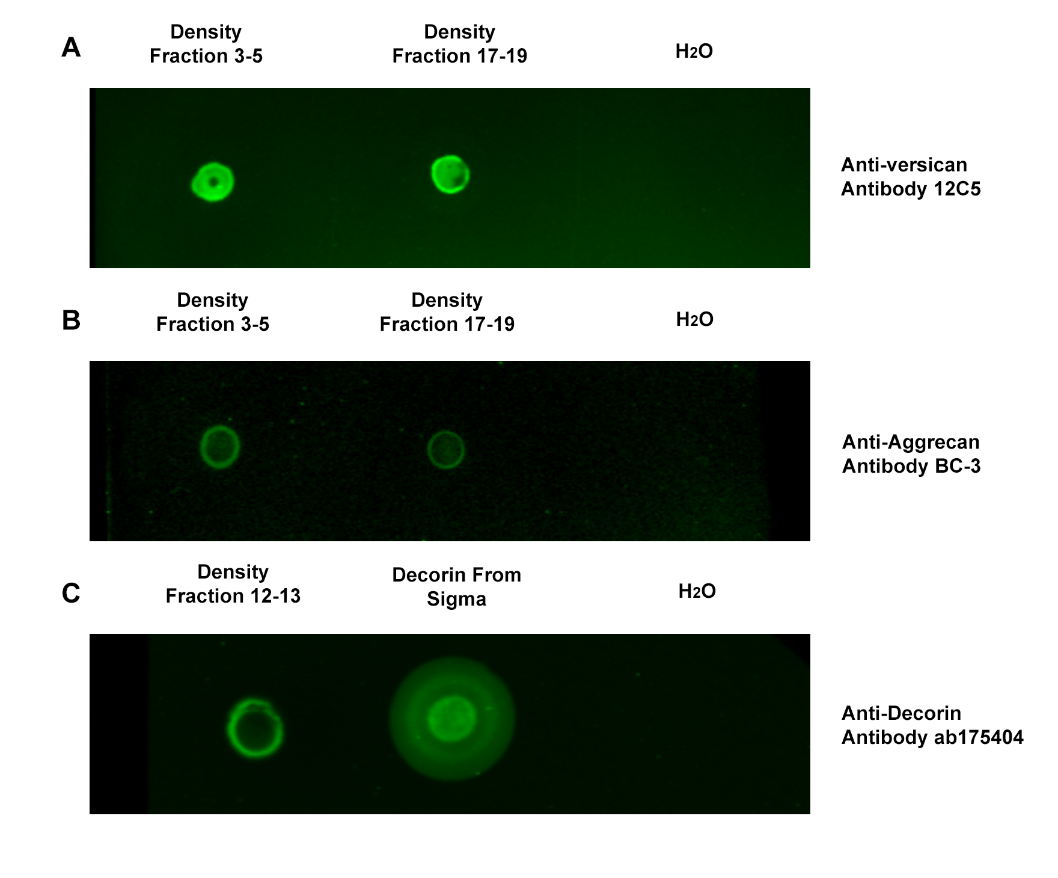


**Supplementary Fig. 2.** Purity of versican preparation. (A) Dot blotting column fractions and staining with versican antibody 12C5 confirmed the presence of versican. (B, C) Dot blots also demonstrated minor contamination of the versican sample with aggrecan (B) and decorin (approximately 0.37 mg/ml decorin in 4.68 mg/ml extracted sample) (C).


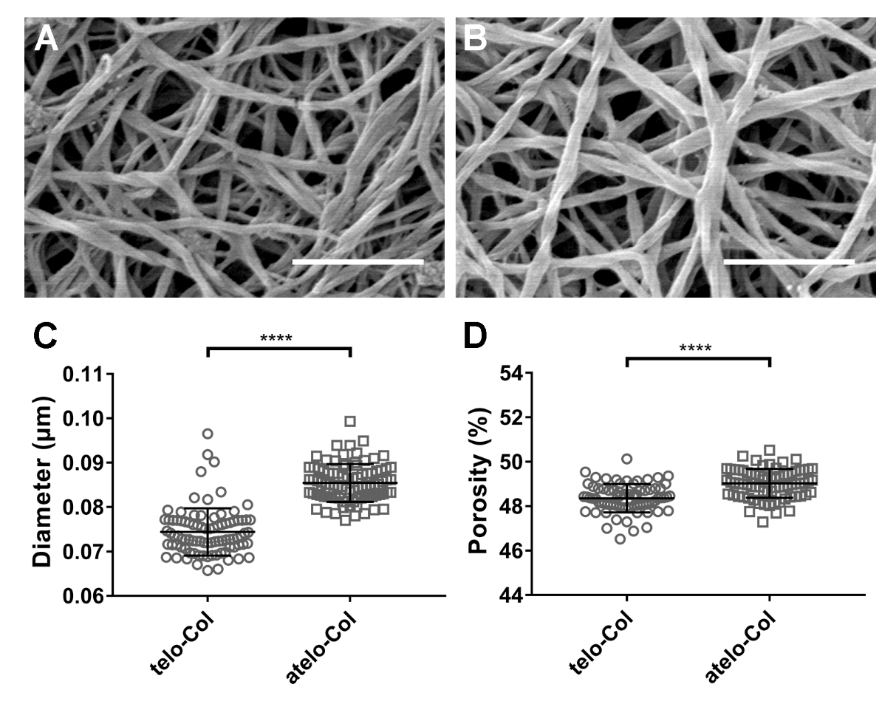


**Supplementary Fig. 3.** The structure of the telo- and atelo-collagen networks with SEM. Atelo-collagen lacks the telo-peptide regions which are the most common sites of covalent crosslinks, and therefore not surprisingly formed a looser network. It also shows thicker fibers compared to telo-collagen. (A, B) SEM imaging of collagen matrices: (A) 1.5 mg/ml telocollagen; (B) 1.5 mg/ml atelocollagen. (C, D) Quantification of fiber diameter and porosity analyzed using DiameterJ. The data show thicker fibers and a looser network for atelocollagen. Three independent experiments were carried out for each condition. 5 images were taken for each gel and measurements taken on 5 random locations for each image. Scale bar = 1µm. Data represent mean ± SD. ****P<0.0001.


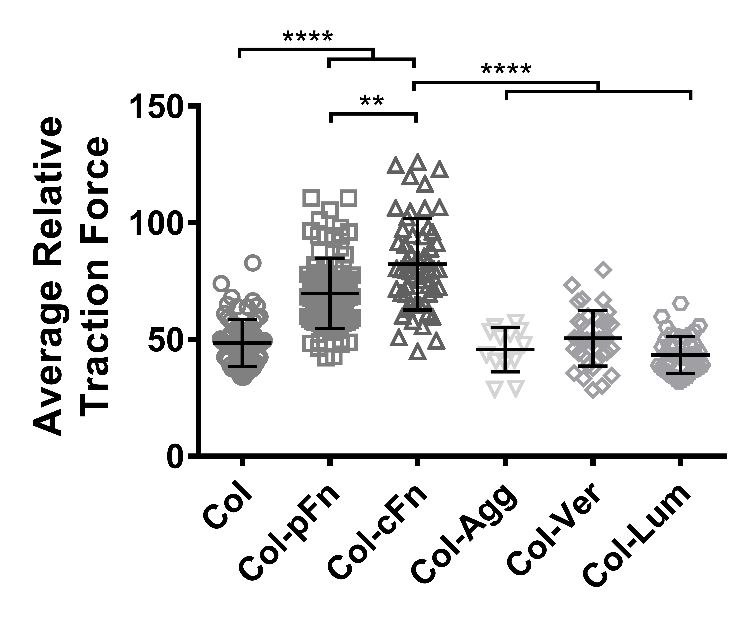


**Supplementary Fig. 4.** Traction force microscopy of NIH 3T3 cells on matrices of various composition. 7.9 kPa polyacrylamide gels were coated with 0.1 mg/ml collagen (Col) mixed with plasma fibronectin (pFn), cellular fibronectin (cFn), aggrecan (Agg), versican (Ver) and lumican (Lum) at 0.1mg/ml. The inclusion of PGs did not alter cellular contractility. In contrast, there was a significant increase with both variants of fibronectin, which are included for comparison. Three independent experiments were carried out for each condition. Data represent mean ± SD. **P<0.01 and ****P<0.0001.


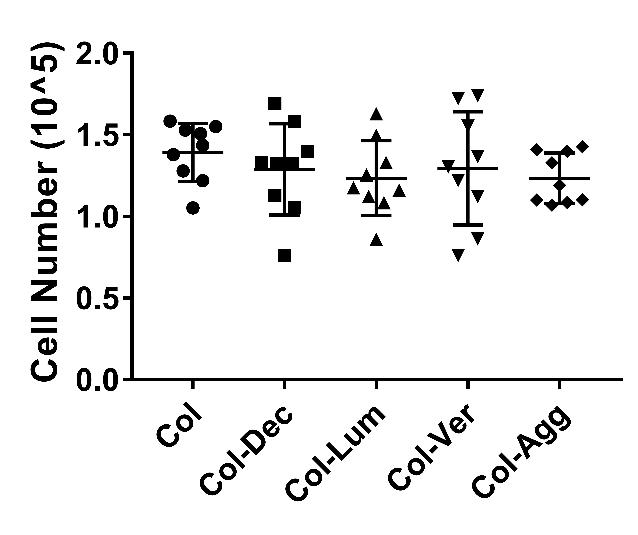


**Supplementary Fig. 5.** 3T3 fibroblasts were cultured in contractile collagen gels with the same proteoglycan manipulations used in the µTUG assay (lumican was tested at 0.05 mg/ml); see Fig. 6. After culture for 24 h, collagen gels were digested with collagenase and the total cell number was counted. We confirmed that the matrix PGs we studied had no influence on proliferation of fibroblasts cultured in collagen gels when compared with collagen alone over 24 h. Three independent experiments were carried out for each condition.
